# Endothelin-1 Downregulates Sulfur Dioxide/Aspartate Aminotransferase Pathway via Reactive Oxygen Species to Promote the Proliferation and Migration of Vascular Smooth Muscle Cells

**DOI:** 10.1155/2020/9367673

**Published:** 2020-01-28

**Authors:** Xiaoyu Tian, Qingyou Zhang, Yaqian Huang, Selena Chen, Chaoshu Tang, Yan Sun, Junbao Du, Hongfang Jin

**Affiliations:** ^1^Department of Pediatrics, Peking University First Hospital, Beijing 100034, China; ^2^Research Unit of Clinical Diagnosis and Treatment of Pediatric Syncope and Cardiovascular Diseases, Chinese Academy of Medical Sciences, Beijing, China; ^3^Division of Biological Sciences, University of California, La Jolla, San Diego, California 92093, USA; ^4^Key Laboratory of Molecular Cardiology, Ministry of Education, Beijing, China; ^5^Department of Physiology and Pathophysiology, Peking University Health Science Centre, Beijing 100091, China

## Abstract

The regulatory mechanisms for proliferation and migration of vascular smooth muscle cells have not yet been clear. The present study was designed to investigate whether and how endothelin-1 (ET-1) impacted the generation of endogenous sulfur dioxide (SO_2_) in rat vascular smooth muscle cell (VSMC) proliferation and migration. Primary VSMCs and purified aspartate aminotransferase (AAT) protein were used in this study. We found that in the presence of ET-1, the expression of PCNA and Ki-67 was upregulated and the migration of VSMCs was promoted, while the AAT activity and SO_2_ levels in VSMCs were reduced without any changes in AAT1 and AAT2 expression. SO_2_ supplementation successfully prevented the ET-1-facilitated expression of PCNA and Ki-67 and the migration of VSMCs. Interestingly, ET-1 significantly increased reactive oxygen species (ROS) production in association with SO_2_/AAT pathway downregulation in VSMCs compared with controls, while the ROS scavenger N-acetyl-L-cysteine (NAC) and the antioxidant glutathione (GSH) significantly abolished the ET-1-stimulated downregulation of the SO_2_/AAT pathway. Moreover, the AAT activity was reduced in purified protein after the treatment for 2 h. However, NAC and GSH blocked the hydrogen peroxide-induced AAT activity reduction. In conclusion, our results suggest that ET-1 results in the downregulation of the endogenous SO_2_/AAT pathway via ROS generation to enhance the proliferation and migration of VSMCs.

## 1. Introduction

The proliferation and migration of vascular smooth muscle cells are the pathophysiological basis of many cardiovascular diseases. A variety of factors are involved in these processes, such as gasotransmitters and vasoactive peptides. The impacts of small vasoactive molecules on vascular proliferation, migration, and remodeling depend on the integrated effects of the molecules [1, 2]. Therefore, it is of great scientific significance to explore the interactions between gasotransmitters and vasoactive peptides and the regulatory mechanisms for vasoactive molecules to understand the possible mechanism underlying vascular smooth muscle cell (VSMC) proliferation and migration.

Endothelin-1 (ET-1) is a plasma protein secreted by vascular endothelial cells that possesses potent vasoconstrictive activity [3]. It induces biological or pathological effects upon binding to the ET_A_ receptor in VSMCs [4]. Previous studies have demonstrated that ET-1 levels are increased in many cardiovascular diseases, such as salt-sensitive hypertension [5] and atherosclerosis [6]. Simultaneously, the proliferation and migration of VSMCs are involved in the development of cardiovascular diseases.

In previous studies, sulfur dioxide (SO_2_) was regarded as an environmental pollutant [7]. In recent years, it was found that the SO_2_ pathway was endogenously generated in cardiovascular tissues. SO_2_ is produced during the metabolism of sulfur-containing amino acids and catalyzed by aspartate aminotransferase 1 (AAT1) and aspartate aminotransferase 2 (AAT2) [8]. At a low concentration, SO_2_ has a variety of physical functions, such as vasodilation and lipid regulation [9]. Animal studies have shown that ET-1 secretion is significantly increased and the SO_2_ content in the serum declines in spontaneously hypertensive rats (SHR) [10, 11]. Recent studies have indicated that ET-1 concentrations are elevated in the lung tissue but SO_2_ levels are decreased in rats with hypoxia-induced pulmonary hypertension [12]. Earlier studies revealed that there were decreased plasma SO_2_ levels and aortic SO_2_ production but increased endothelial ET-1 levels in atherosclerosis [13, 14]. The results indicated that there might be an interaction between ET-1 and SO_2_ in cardiovascular diseases. However, the impact of ET-1 on the endogenous SO_2_ pathway and the subsequent effects on the proliferation and migration of vascular smooth muscle cells remain unclear.

Reactive oxygen species (ROS) are by-products of normal oxygen metabolism [15]. They are atoms or molecules that possess one or more unpaired electrons in the outer orbit, including the hydroxyl radical (^·^OH), hydrogen peroxide (H_2_O_2_), superoxide anion (O_2_^·-^) and peroxynitrite (ONOO^·-^) [16]. It has been known for years that vascular and cardiac tissues are rich sources of ROS, and smooth muscle cells and fibroblasts produce the majority of O_2_^·-^ found in the normal vessel wall [17]. ROS are implicated in the pathogenesis of vascular injury diseases [18]. It was reported that plasma ET-1 levels were significantly increased in patients with pulmonary hypertension and that increased ET-1 binding to smooth muscle cell receptors resulted in persistent elevation of ROS levels, further aggravating vascular damage [19]. A recent report suggested that pulmonary remodeling was associated with increased ROS production in a lamb pulmonary hypertension model induced by catheter ligation [20]. ROS within cells act as secondary messengers in intracellular signaling cascades, and SO_2_ is highly affected by the redox state *in vivo* [21]. Therefore, the present study was undertaken to examine whether ET-1 regulates the generation of endogenous SO_2_ in VSMCs, affecting the proliferation and migration of VSMCs and the possible mechanisms associated with ROS production.

## 2. Materials and Methods

### 2.1. Cell Culture and Grouping

In cell experiments *in vitro*, primary VSMCs were harvested from male Wistar rats (Vital River, Beijing, China) weighing 180-200 g. The thoracic aorta was stripped of the adventitia and aseptically transferred into culture medium. Primary VSMCs were isolated and cultured according to the method used by Chi et al. [22]. The cells were cultured in Dulbecco's modified Eagle's medium (DMEM) (Gibco, USA) supplemented with 10% FBS in an atmosphere of 5% CO_2_ at 37°C. The media were changed to serum-free DMEM for 24 h when the VSMCs were grown to 70%-80% confluence. This study was approved by the Animal Ethics Committee of Peking University First Hospital (EC Approval No. J 201858) and was conducted in strict accordance with applicable regulations of the Animal Ethics Committee of Peking University.

Cells were divided into the control and experimental groups. ET-1 at a concentration of 10^−8^ M, 10^−7^ M, and 10^−6^ M was added to the experimental group as previously described [23–26], and for the control group, the same volume of a sterile PBS buffer solution was added. To assess the effects on the proliferation and migration of VSMCs, cells were divided into the following groups: control group, 10^−6^ M ET-1 group, 100 *μ*M SO_2_ group, and ET-1+SO_2_ group.

To investigate the effects of ET-1 on the endogenous SO_2_ pathway and its mechanisms in VSMCs, cells were divided into the following groups: control group, 10^−6^ M ET-1 group, N-acetyl-L-cysteine (NAC) group, NAC+10^−6^ M ET-1 group, glutathione (GSH) group, and GSH+10^−6^ M ET-1 group. The concentrations of NAC and GSH were 5 mM and 10 mM, respectively. In purified protein experiments, purified AAT protein was divided into the control group, 200 *μ*M H_2_O_2_ group, 200 *μ*M H_2_O_2_+5 mM NAC group, and 200 *μ*M H_2_O_2_+10 mM GSH group.

### 2.2. *In Situ* Fluorescence Measurement of SO_2_

Endogenous SO_2_ in primary VSMCs was observed using a fluorescent probe (provided by Professor Kun Li and Xiaoqi Yu, Sichuan University, Sichuan, China) [27]. After drug stimulation for 48 h, an SO_2_ fluorescent probe working fluid (10 *μ*M) was added to the medium of cells and incubated for 30 min at 37°C. The cells were washed 3 times with 0.01 M PBS. The cells were fixed in 4% paraformaldehyde for 15 to 20 min at room temperature and rinsed 3 times in 0.01 M PBS for 5 min each time. The cells were detected as blue fluorescence by confocal microscopy.

### 2.3. Western Blotting

Primary VSMCs were seeded into 6-well plates. After drug treatment for 48 h, cell extracts were resolved on an SDS polyacrylamide gel and blotted on NC membranes. Nonspecific binding was blocked by an incubation in a 5% milk blocking buffer. The NC membranes were incubated overnight at 4°C with the following primary antibodies: anti-AAT1 (1 : 1000; Sigma-Aldrich, USA), anti-AAT2 (1 : 1000; Sigma-Aldrich, USA); anti-PCNA (1 : 1000, Anbo Biotechnology, USA), anti-Ki-67 (1 : 500; Zhongshan Jinqiao, China), and anti-GAPDH (1 : 4000; Shanghai Kangcheng, China). The membranes were then washed and hybridized for 1 h at room temperature with the following secondary antibodies: HRP-conjugated goat anti-rabbit (Cell Signaling Technology, USA), HRP-conjugated goat anti-mouse (Cell Signaling Technology, USA), and HRP-conjugated rabbit anti-goat (Santa Cruz, USA). Immunoreactions were visualized using an enhanced chemiluminescence detection kit.

### 2.4. Cell Scratch-Wound Assay

Primary VSMCs were seeded into a cell migration chamber (IBIDI, Germany). After the cells adhered to the chamber, the medium was replaced with serum-free medium for 24 h to synchronize the cells. Then, the migration chamber was removed, and the length of the cell scratch was immediately photographed under a microscope. Then, 100 *μ*M SO_2_ and 10^−6^ M ET-1 were added to the medium, and cell migration was determined again at 48 h.

### 2.5. AAT Activity Analysis

After drug stimulation for 48 h, primary VSMCs were harvested and lysed in PBS. A portion of the isolated protein was used to detect the protein concentration by Bradford's method, and the remaining protein was used to detect AAT activity. AAT activity was tested by using an AAT activity microplate test kit (Nanjing Jiancheng Bioengineering Institute, China) according to the manufacturer's instructions.

### 2.6. Determination of ROS

Cells were seeded into 8-well chambers and allowed to adhere for at least 24 h. The cells were grown to 60%-70% confluence and incubated in serum-free DMEM supplemented with ET-1, NAC, and GSH as described. After 48 h, 10 *μ*M dihydroethidium (DHE) was added to the medium, and the cells were incubated for an additional 30 min. The cells were then washed with PBS 3 times. DHE-stained cells were observed as red fluorescence by confocal microscopy. The average fluorescence intensities were quantified using Image J.

### 2.7. Data Analysis

Data were expressed as mean ± SEM, and data analysis was performed with SPSS 20.0 software. When the data were normally distributed, comparisons among multiple groups were made by one-way ANOVA followed by the LSD test for post hoc comparisons as appropriate. Otherwise, Dunnett's T3(3) test was used for comparisons. *P* < 0.05 was considered significant.

## 3. Results

### 3.1. ET-1 Downregulates the SO_2_/AAT Pathway in Primary VSMCs

**Figure 1:**
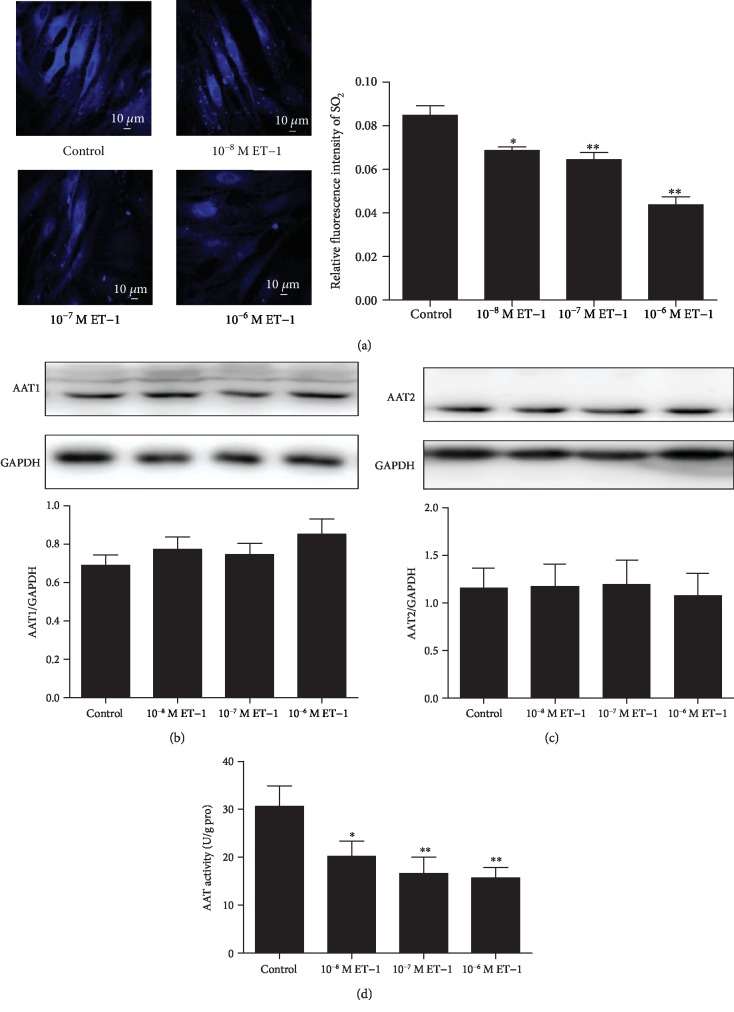
ET-1 downregulated the SO_2_/AAT pathway in primary VSMCs. *In situ* fluorescent staining (blue) detection of endogenous SO_2_ in primary VSMCs and quantified average fluorescence intensity by Image J. Fluorescent signal intensity reflects SO_2_ levels (a). Protein expression of AAT1 and AAT2 in primary VSMCs (b, c). AAT activity in VSMCs (d). Results are expressed as mean ± SEM (*n* = 3‐6). ^∗^*P* < 0.05, ^∗∗^*P* < 0.01 vs. control. AAT1: aspartate aminotransferase 1; AAT2: aspartate aminotransferase 2; ET-1: endothelin-1; VSMCs: vascular smooth muscle cells.

Cultured primary VSMCs were treated with different concentrations of ET-1 (10^−8^, 10^−7^, or 10^−6^ M) for 48 h. We found that compared with that of the control, the fluorescence signal for the SO_2_ content of the treated VSMCs gradually weakened (all *P* < 0.05) in the 10^−8^, 10^−7^, and 10^−6^ M ET-1 groups (Figure 1(a)).

Next, we determined whether AAT activity and expression were affected by ET-1. There was no significant difference (all *P* > 0.05) in the primary VSMC expression of AAT between the control VSMCs and VSMCs treated with different concentrations of ET-1 (10^−8^, 10^−7^, or 10^−6^ M) (Figures 1(b) and 1(c)). However, the AAT enzyme activity was notably reduced (all *P* < 0.05), respectively, in all the cells treated with ET-1 (10^−8^ M, 10^−7^ M, and 10^−6^ M) compared with the controls (Figure 1(d)).

### 3.2. Supplementation with SO_2_ Abolished ET-1-Induced VSMC Proliferation and Migration

**Figure 2:**
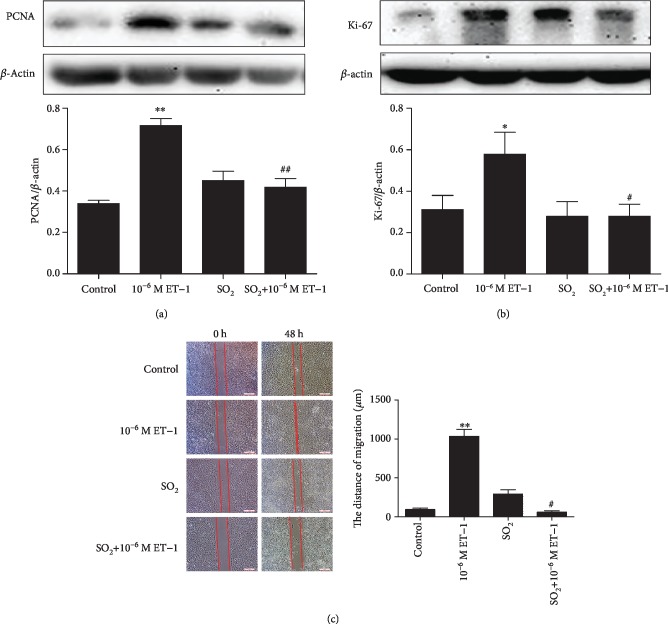
SO_2_ reversed the proliferation and migration of primary VSMCs induced by ET-1. Western blot to detect the protein expression of PCNA and Ki-67 in primary VSMCs (a, b). Cell scratch-wound assay was used to detect the distance of primary VMSC migration (c). Results are expressed as mean ± SEM (*n* = 6‐10). ^∗^*P* < 0.05, ^∗∗^*P* < 0.01 vs. control; ^#^*P* < 0.05, ^##^*P* < 0.01 vs. 10^−6^ M ET-1. ET-1: endothelin-1; SO_2_: sulfur dioxide; PCNA: proliferating cell nuclear antigen; Ki-67: antigen Ki-67.

The results showed that compared with the control group, the 10^−6^ M ET-1 group exhibited significantly enhanced expression of PCNA and Ki-67 (*P* < 0.05, Figures 2(a) and 2(b)) and a narrower wound width (*P* < 0.01, Figure 2(c)). However, pretreatment of the cells with 100 *μ*M SO_2_ successfully abolished the increased PCNA and Ki-67 protein expression and cell migration induced by ET-1 in primary VSMCs.

### 3.3. Involvement of ROS in the ET-1-Induced Reduction in the SO_2_/AAT Pathway

**Figure 3:**
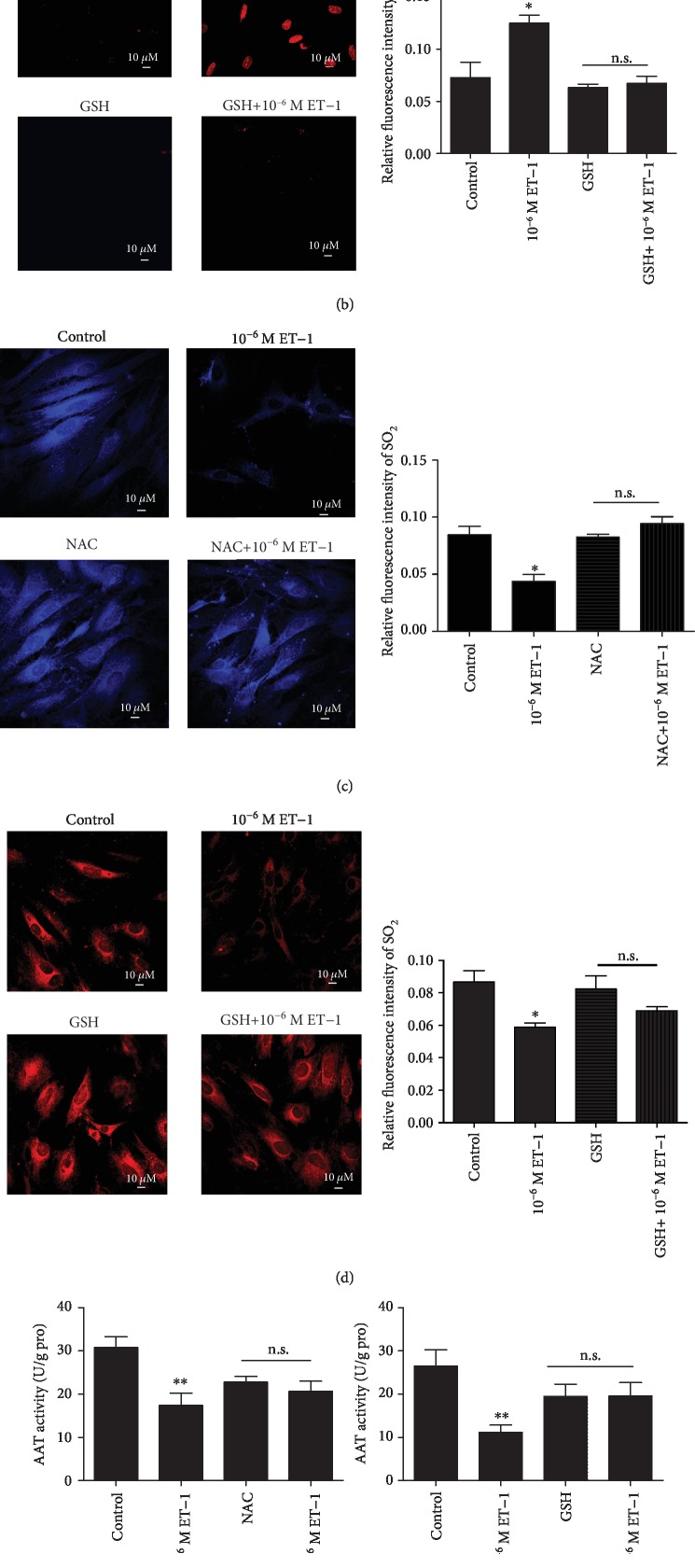
Involvement of ROS in the ET-1-reduced SO_2_/AAT pathway. *In situ* fluorescent staining (red) detection of ROS in primary VSMCs and quantified average fluorescence intensity by ImageJ. Fluorescent signal intensity reflects ROS levels (a, b). *In situ* fluorescent staining (red and blue) detection of endogenous SO_2_ in primary VSMCs and quantified average fluorescence intensity by Image J. Fluorescent signal intensity reflects SO_2_ levels (c, d). AAT activity in VSMCs and purified protein (e, f). Results are expressed as mean ± SEM (*n* = 3‐6). ^∗^*P* < 0.05, ^∗∗^*P* < 0.01 vs. control; ^##^*P* < 0.01 vs. 200 *μ*m H_2_O_2_. n.s. stands for no significant difference. ROS: reactive oxygen species; H_2_O_2_: hydrogen peroxide; ET-1: endothelin-1; NAC: N-acetyl-L-cysteine; GSH: glutathione; VSMCs: vascular smooth muscle cells.

To gain insight into the mechanisms by which ET-1 reduces endogenous SO_2_ generation, we detected the intracellular ROS and SO_2_ levels and AAT activity in primary VSMCs. As expected, compared with the controls, in the 10^−6^ M ET-1-treated cells, the red fluorescence signal intensity indicative of ROS in primary VSMCs was markedly increased (*P* < 0.05), whereas the level of SO_2_ and AAT activity was decreased (*P* < 0.05, Figures 3(a)–3(e)). The presence of the ROS scavenger NAC successfully abolished the downregulation of the SO_2_ level and the reduced AAT activity associated with the ROS elevation. Similarly, pretreatment with another antioxidant, GSH, also significantly abrogated the ET-1-induced reduction in the SO_2_ content and the reduced AAT activity associated with the ROS augmentation (Figures 3(a)–3(e)).

To further confirm the direct effect of ROS on AAT activity, we measured the AAT activity of purified AAT protein. The data showed that AAT activity was significantly reduced (*P* < 0.01) in the presence of 200 *μ*M H_2_O_2_ compared with control treatment. In the presence of 5 mM NAC and 10 mM GSH, the H_2_O_2_-induced reduction in the AAT activity of purified AAT protein was blocked (*P* < 0.01, Figure 3(f)).

## 4. Discussion

**Figure 4:**
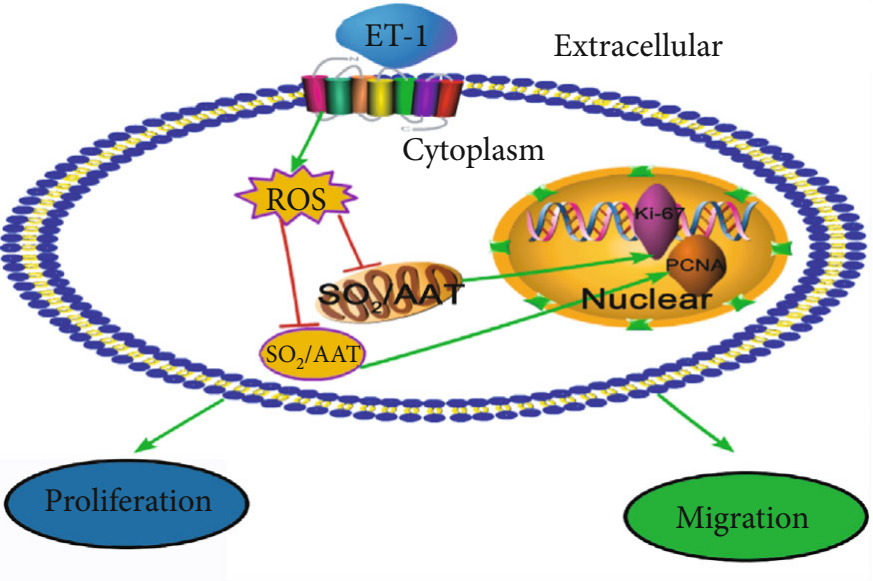
Schematic drawing depicting relations among ET-1, ROS, and the SO_2_/AAT pathway.

In the present study, we detected changes in the VSMC-derived SO_2_ production induced by ET-1. We found that ET-1 reduced SO_2_ generation by decreasing the activity of AAT without influencing AAT1 and AAT2 expression and that the ET-1-mediated reduction in the SO_2_ level participated in the excessive proliferation and migration of VSMCs (Figure 4).

We further explored the molecular mechanisms by which ET-1 triggers a reduction in AAT activity. In recent years, it has been shown that ROS acts as a secondary messenger and participates in multiple signal transduction pathways in cells to exert biological effects. ROS plays a major role in the pathogenesis of various cardiovascular diseases, including hypertension and atherosclerosis [28]. Most of these studies showed a possible relationship between ET-1 ROS and cardiac hypertrophy [29]. There was also evidence of a relationship among ET-1, ROS and VSMCs. In a study investigating pulmonary hypertension, plasma ET-1 levels were significantly elevated, and the increased ET-1 level was associated with an elevation in the ROS level [30]. ET-1 increases ROS production in VSMCs and functions as a critical mediator in ET-1-induced signaling events in association with growth-promoting proliferative and hypertrophic pathways in VSMCs [31]. In previous studies, some mechanisms were reported to be involved in the effects of ET-1 on ROS generation. Touyz et al. found that the mitochondrial inhibitor, but not the NADPH oxidase inhibitor, attenuated the ET-1-induced ROS generation and the subsequent MAPK pathway activation in human vascular smooth muscle cells, suggesting that mitochondria might be an important source of ET-1-induced ROS [23], while it was found that ET-1 activated NADPH oxidase subunit Nox5 and therefore induced O_2_^·-^ generation in human endothelial cells [32]. Moreover, xanthine oxidase was also found to be involved in the ET-1-induced generation of ROS in the aorta and resistance arteries in rats [33]. Furthermore, the ET_A_ receptor not the ET_B_ receptor was found to be coupled to O_2_^·-^ and H_2_O_2_ production induced by ET-1 in pulmonary vascular smooth muscle cells [34, 35] or in cardiac fibroblasts [36], while the ET_B_ receptor antagonist prevented the ET-1-enhanced ROS generation in human umbilical vein endothelial cells [37]. SO_2_ is a gasotransmitter that is highly sensitive to oxidative damage and is vulnerable to the steady-state changes of redox reactions. Therefore, we further explored whether ROS was the key link to the reduction in SO_2_ generation mediated by ET-1.

Our present study showed that ET-1 could induce a reduction in SO_2_ generation associated with ROS level elevation in VSMCs, and this effect was abolished by the oxidative stress scavenger NAC and the antioxidant GSH, demonstrating that NAC and GSH can mediate a reversal of the reductions in SO_2_ content and AAT activity induced by ET-1. To further study the direct role of ROS in AAT activity, purified AAT protein was stimulated with H_2_O_2_, and we found that ROS lowered the activity of AAT, but NAC and GSH abrogated this effect. The results indicated that ROS was involved in the ET-1-triggered reduction in SO_2_ generation in VSMCs (Figure 4).

A previous study showed that H_2_S acting as a gasotransmitter can inhibit the proliferation of vascular smooth muscle cells [38–40]. Some research reported that SO_2_ could inhibit the proliferation and migration of myocardial fibroblasts [41]. In a study of hypoxia-induced pulmonary arteriolar remodeling, it was found that SO_2_ significantly attenuated the interstitial thickening and prominent media hypertrophy of pulmonary arteries [42]. Therefore, we detected whether SO_2_ is involved in the excessive proliferation and migration of vascular smooth muscle cells. Our results showed ET-1-induced VSMC proliferation, as demonstrated by the increase in PCNA and Ki-67 expression and the wound width narrowing. However, SO_2_ could prevent the ET-1-induced proliferation and migration of VSMCs.

Our research provides evidence of the interactions between peptides and gasotransmitters, which will help in the understanding of the mechanisms by which vascular regulation by vasoactive small molecules depends on the integrated effects of these molecules as a complex network. These results are also significant for understanding the pathogenesis of vascular injury diseases, further deepening the significance of endogenous SO_2_ in vascular injury diseases mainly characterized by smooth muscle cell proliferation and migration. However, in the future, further studies are needed to explore the molecular mechanism for the ROS-induced reduction in AAT activity and its significance in animal models.

## 5. Conclusion

The present study indicated that ET-1 downregulated the endogenous SO_2_/AAT pathway, which is involved in the formation of proliferation and migration of VSMCs. The excessive ROS production mediated the ET-1-induced reduction of SO_2_/AAT pathway. The above findings would be of great value in the understanding of the role of the endogenous SO_2_/AAT pathway in the mechanisms for vascular diseases.

## Data Availability

The data used to support the findings of this study are available from the corresponding authors upon request.
